# Estrogens influence female itch sensitivity via the spinal gastrin-releasing peptide receptor neurons

**DOI:** 10.1073/pnas.2103536118

**Published:** 2021-07-26

**Authors:** Keiko Takanami, Daisuke Uta, Ken Ichi Matsuda, Mitsuhiro Kawata, Earl Carstens, Tatsuya Sakamoto, Hirotaka Sakamoto

**Affiliations:** ^a^Ushimado Marine Institute, Graduate School of Natural Science and Technology, Okayama University, Setouchi 701-4303, Japan;; ^b^Anatomy and Neurobiology, Kyoto Prefectural University of Medicine, Kyoto 602-8566, Japan;; ^c^Mouse Genomics Resources Laboratory, National Institute of Genetics, Department of Genetics, SOKENDAI (The Graduate University for Advanced Studies), Mishima 411-8540, Japan;; ^d^Department of Neurobiology, Physiology and Behavior, University of California, Davis, CA 95616;; ^e^Department of Applied Pharmacology, Faculty of Pharmaceutical Sciences, University of Toyama, Toyama 930-0194, Japan;; ^f^School of Health Science, Bukkyo University, Kyoto 604-8418, Japan

**Keywords:** itch, estrogens, histamine, gastrin-releasing peptide receptor, spinal cord

## Abstract

There are sex differences in somatosensory sensitivity. Circulating estrogens appear to have a pronociceptive effect that explains why females are reported to be more sensitive to pain than males. Although itch symptoms develop during pregnancy in many women, the underlying mechanism of female-specific pruritus is unknown. Here, we demonstrate that estradiol, but not progesterone, enhances histamine-evoked scratching behavior indicative of itch in female rats. Estradiol increased the expression of the spinal itch mediator, gastrin-releasing peptide (GRP), and increased the histamine-evoked activity of itch-processing neurons that express the GRP receptor (GRPR) in the spinal dorsal horn. The enhancement of itch behavior by estradiol was suppressed by intrathecal administration of a GRPR blocker. In vivo electrophysiological analysis showed that estradiol increased the histamine-evoked firing frequency and prolonged the response of spinal GRP-sensitive neurons in female rats. On the other hand, estradiol did not affect the threshold of noxious thermal pain and decreased touch sensitivity, indicating that estradiol separately affects itch, pain, and touch modalities. Thus, estrogens selectively enhance histamine-evoked itch in females via the spinal GRP/GRPR system. This may explain why itch sensation varies with estrogen levels and provides a basis for treating itch in females by targeting GRPR.

## Significance

Many women exhibit a dramatic increase in itch during pregnancy, but the underlying mechanism is unknown. Here, we demonstrate that the female sex steroid hormone estradiol, but not progesterone, enhances itch-related scratching behavior in female rats elicited by histamine, the prototypical itch mediator in humans. This is associated with an enhancement in histamine-evoked activity of a subset of spinal dorsal horn neurons that express a neuropeptide receptor, gastrin-releasing peptide receptor (GRPR), that was previously shown to be involved in spinal cord processing of itch. These findings may account for why itch sensation varies with estrogen levels and provide a basis for treating histamine-related itch diseases in females by targeting GRPR.

Itch and pain warn the body of potential tissue damage and are indispensable to survival. The threshold and intensity of itch and pain are not absolute but depend on various environmental and psychological factors such as stress, mood, and anxiety (1–3). Female-specific pruritus occurs during pregnancy and menopause when circulating female hormones dramatically fluctuate (4, 5). Approximately 20% of pregnant women have itch symptoms during pregnancy (6), such as pruritic urticarial papules and plaques of pregnancy, intrahepatic cholestasis of pregnancy, and pemphigoid gestationis. Female patients with atopic dermatitis often experience atopic eruption and cutaneous deterioration associated with their pregnancy (7–9). Although these itch symptoms impair female quality of life, the mechanism is still unknown. Histamine is an important peripheral itch mediator. Once released from mast cells activated by irritant stimuli and allergens, histamine induces not only inflammation but also itch triggered by the excitation of a subset of unmyelinated C fibers (10). Centrally, neurons expressing the gastrin-releasing peptide (GRP) and its receptor (GRPR) play a critical role in the spinal cord transmission of itch signals independent of pain (11–15). In the present study, we focused on the female sex hormones as candidates for the cause of itch in women. We discovered that female itch sensitivity is induced by estrogens via the spinal GRP/GRPR system in rats.

## Results

**Fig. 1.**
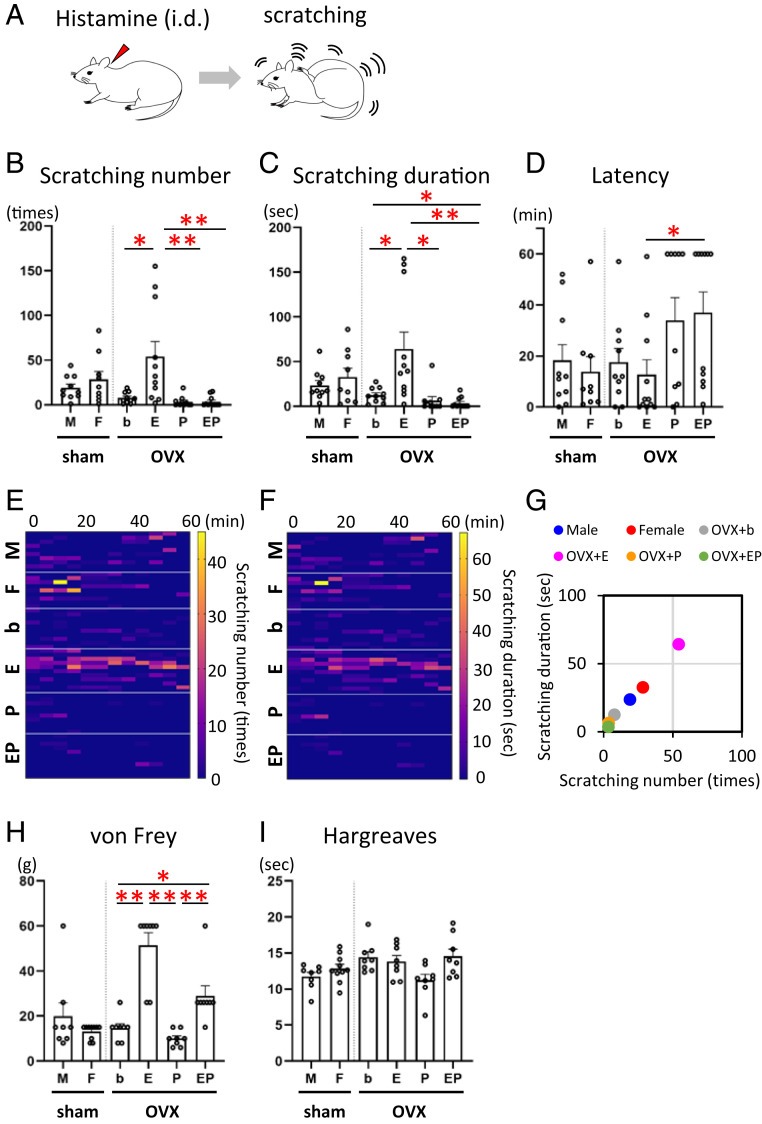
Estrogens enhance histamine-evoked itch behavior but decrease pain behavior. (*A*) Schematic showing histamine-evoked scratching behavior. (*B*–*D*) Scratching behavior is monitored for 60 min after intradermal injection of histamine (3 mg/100 μL). (*B*) The number of histamine-evoked scratch bouts was markedly increased in the estrogen treatment group among OVX treatment groups. *n* = 9 to 11/group; sex difference analysis: Student’s *t* test: T (17) = 0.963, *P* = 0.349, OVX group comparative analysis: Kruskal–Wallis test: *H* = 20.085, df = 3, *P* = 0.000; Bonferroni-corrected Mann–Whitney *U* post hoc test: U [19] = 17.000, Z = 2.679, *P* = 0.006, b versus E; U (19) = 8.000, Z = 3.334, *P* = 0.000, E versus P; U (20) = 8.000, Z = 3.483, *P* = 0.000, E versus EP. (*C*) The duration of histamine-evoked scratch bouts was markedly increased in the estrogen treatment group. Sex difference analysis: Welch’s *t* test: T (12.112) = 0.800, *P* = 0.439, OVX group comparative analysis: Kruskal–Wallis test: *H* = 22.085, df = 3, *P* = 0.000; Bonferroni-corrected Mann–Whitney *U* post hoc test: U (19) = 17.000, Z = 2.676, *P* = 0.006, b versus E; U (19) = 15.000, Z = 2.849, *P* = 0.004, b versus EP; U (19) = 10.000, Z = 3.190, *P* = 0.001, E versus P; U (20) = 5.000, Z = 3.681, *P* = 0.000, E versus EP. (*D*) The latency to histamine-evoked scratching was shorter in the estrogen treatment group than the EP treatment group. Sex difference analysis: Mann–Whitney *U* test: U (17) = 41.000, Z = 0.327, *P* = 0.780, OVX group comparative analysis: Kruskal–Wallis test: *H* = 8.687, df = 3, *P* = 0.034; Bonferroni-corrected Mann–Whitney *U* post hoc test: U (20) = 20.500, Z = 2.661, *P* = 0.007, E versus EP. (*E*) Heat map showing the number of histamine-evoked scratch bouts every 5 min for each treatment group. (*F*) Heat map showing the total time of histamine-evoked scratching every 5 min for each treatment group. (*G*) Correlation between the number of scratches and the total scratching time. The value is the average of each group. (*H*) Mechanical sensitivity measured by paw withdrawal threshold upon exposure to von Frey filaments was significantly decreased in the estradiol-treated rats (*n* = 8 to 10/group; sex difference analysis: Mann–Whitney *U* test: U (16) =34.500, Z = 0.539, *P* = 0.633, OVX group comparative analysis: Kruskal–Wallis test: *H* = 24.201, df = 3, *P* = 0.000; Bonferroni-corrected Mann–Whitney *U* post hoc test: U (14) = 1.000, Z = 3.410, *P* = 0.000, b versus E; U (14) = 6.500, Z = 2.880, *P* = 0.005, b versus EP; U (14) = 0.000, Z = 3.470, *P* = 0.000, E versus P; U (14) = 1.000, Z = 3.366, *P* = 0.000, P versus EP). (*I*) Responses to noxious thermal stimulation measured by the paw withdrawal latency (Hargreaves) test were indistinguishable between the groups (*n* = 8 to 10/group; sex difference analysis: Student’s *t* test: T (16) = 1.303, *P* = 0.211, OVX group comparative analysis: one-way ANOVA: F (3, 28) = 3.227, *P* = 0.037; post hoc Tukey’s tests do not show any significance between each group). Kruskal–Wallis test with Bonferroni-corrected Mann–Whitney *U* post hoc analysis in *B*, *C*, *D*, and *H*; *P < 0.0083, **P < 0.001 for Bonferroni-corrected Mann–Whitney *U* post hoc test following Kruskal–Wallis test among four OVX groups. Data are mean ± SEM; i.d., intradermal injection; M, male; F, female; OVX, ovariectomy; b, blank; E, estradiol; P, progesterone; EP, estradiol and progesterone treatment group. See also *SI Appendix*, Figs. S1–S3.

To determine the effects of female sex hormones on itch sensitivity, we first investigated histamine-evoked hind paw scratching as a marker of itch behavior (Fig. 1*A*) in male and female Wistar rats as well as in bilaterally ovariectomized (OVX) females implanted with a blank (b) capsule as a control (OVX + b), OVX females treated with a physiological level of 17β-estradiol (E) (OVX + E), OVX females treated with a physiological level of progesterone (P) (OVX + P), and OVX females treated with both estradiol and progesterone (OVX + EP) (*SI Appendix*). Estradiol, but not progesterone, replacement strikingly induced a significant elevation in the number of scratch bouts (Fig. 1 *B* and *E* and *SI Appendix*, Fig. S1: Kruskal–Wallis post hoc test: *P < 0.0083, b versus E; **P < 0.001, E versus P and E versus EP) and prolonged the duration (Fig. 1 *C* and *F*: Kruskal–Wallis post hoc test: *P < 0.0083; b versus E, b versus EP, E versus P, **P < 0.001; E versus EP) of histamine-evoked scratching behavior. Estradiol also shortened the latency of histamine-evoked scratching (Fig. 1*D*: Kruskal–Wallis post hoc test: *P < 0.0083, E versus EP). A significant correlation was observed between the number of scratch bouts and the total duration of scratching/60 min in all groups (Fig. 1*G*: Pearson’s correlation coefficient test: Male, *r* = 0.906, *P* = 0.000; Female, *r* = 0.889, *P* = 0.001; OVX + b, *r* = 0.960, *P* = 0.000: OVX + E, *r* = 0.990, *P* = 0.000; OVX + P, *r* = 0.975, *P* = 0.000; OVX + EP, *r* = 0.941, *P* = 0.000). We additionally observed a sex difference as well as estrogen enhancement of scratching elicited by another itch mediator, chloroquine (CQ) (*SI Appendix*, Fig. S2 *A*–*H*). Intradermal injection of saline vehicle did not elicit significant scratching in any OVX group (*SI Appendix*, Fig. S3 *A*–*C*). In contrast, estradiol reduced sensitivity to innocuous von Frey mechanical stimuli (Fig. 1*H*: Kruskal–Wallis post hoc test: *P < 0.0083, b versus EP, **P < 0.001; b versus E, E versus P, P versus EP). In addition, estradiol did not affect the thermal pain sensitivity (Fig. 1*I*). In summary, estradiol enhanced histamine- and CQ-evoked scratching behavior in OVX females while reducing mechanosensitivity.

**Fig. 2.**
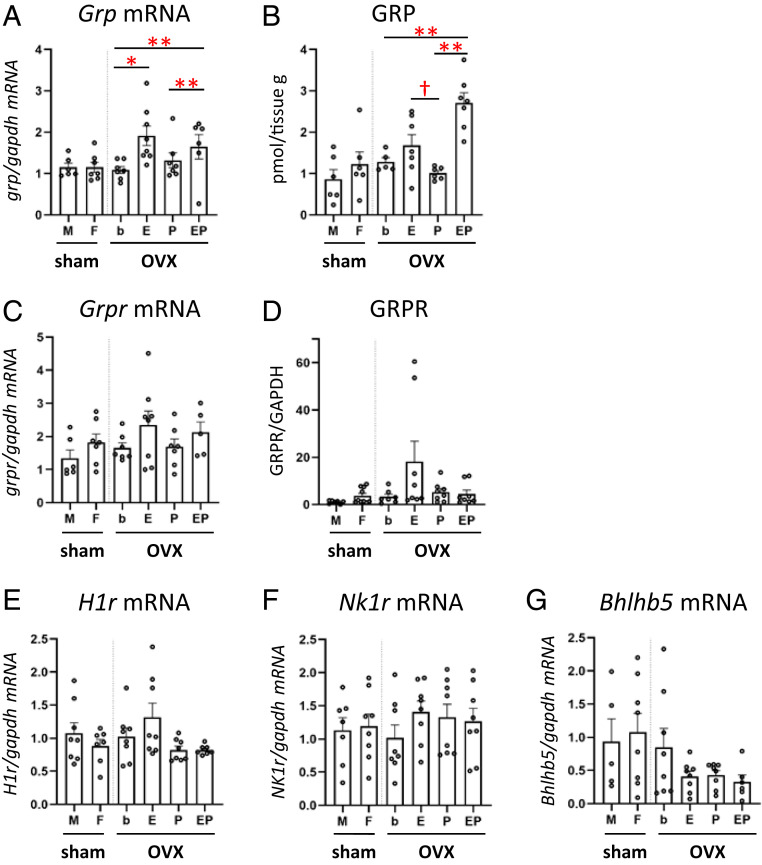
Effects of hormone replacement on expression of itch- and pain-related molecules in the spinal dorsal horn. (*A*) Estradiol replacement significantly increased the level of *Grp* mRNA. *n* = 6 to 8/group; sex difference analysis: Student’s *t* test: T (11) = 0.003, *P* = 0.998, OVX group comparative analysis: one-way ANOVA: F (3, 22) = 7.724, *P* = 0.001, post hoc Gomes–Howell test: *P* = 0.039, b versus E; *P* = 0.004, b versus EP; *P* = 0.008, P versus EP. (*B*) The EP treatment group showed the increase of GRP protein level. *n* = 5 to 7/group; sex difference analysis: Student’s *t* test: T (10) = 0.966, *P* = 0.357, OVX group comparative analysis: one-way ANOVA: F (3, 21) = 13.267, *P* = 0.000, post hoc Gomes–Howell test: *P* = 0.003, b versus EP; *P* = 0.001, P versus EP. The estradiol treatment showed significantly higher GRP levels than the progesterone treatment group [Welch’s *t* test: T (6.678) = 2.504, *P* = 0.042]. (*C*) The expression level of *Grpr* mRNA did not differ between the groups. *n* = 5 to 8/group; sex difference analysis: Mann–Whitney *U* test: U (11) =12.500, Z = 1.216, *P* = 0.234, OVX group comparative analysis: one-way ANOVA: F (3, 23) = 1.271, *P* = 0.308. (*D*) There was no difference in the expression level of GRPR protein between the groups. *n* = 6 to 10/group; sex difference analysis: Mann–Whitney *U* test: U (15) = 20.000, Z = 1.464, *P* = 0.161, OVX group comparative analysis: Kruskal–Wallis test: *H* = 4.123, df = 3, *P* = 0.248). (*E*–*G*) There were no significant differences in the expression levels of *H1r* mRNA in the DRG [*n* = 7 to 8/group; sex difference analysis: Student’s *t* test: T (13) = 1.002, *P* = 0.355, OVX group comparative analysis: one-way ANOVA: F (3, 27) = 2.998, *P* = 0.048; post hoc Gomes–Howell tests do not show any significance between each group] and *NK1r* [*n* = 7 to 8/group; sex difference analysis: Student’s *t* test: T (13) = 0.227, *P* = 0.824, OVX group comparative analysis: Kruskal–Wallis test: *H* = 2.754, df = 3, *P* = 0.431) and *Bhlhb5* [*n* = 5 to 8/group; sex difference analysis: Student’s *t* test: T (11) = 0.314, *P* = 0.760, OVX group comparative analysis: one-way ANOVA: F (3, 26) = 1.989, *P* = 0.140] mRNAs in the spinal dorsal horn. The *y*-axis in *A*, *C*, and *E* through *G* shows expression levels (fold change) relative to *Gapdh* mRNA in the cervical spinal dorsal horn (*A*, *C*, *F*, and *G*) and DRG (*E*) and GAPDH protein in the cervical spinal dorsal horn (*D*). *P < 0.05, **P < 0.01, ^†^*P* < 0.05, one-way ANOVA with Tukey post hoc analysis. Data are mean ± SEM. M, male; F, female; OVX, ovariectomy; b, blank; E, estradiol; P, progesterone; EP, estradiol and progesterone both treatment group; DH, spinal dorsal horn; DRG, dorsal root ganglion; NK1R, neurokinin 1 receptor; Bhlhb5, basic helix–loop–helix b5.

We next addressed whether estrogens affect the expression of itch and pain mediators in the spinal somatosensory system. GRP- and GRPR-expressing neurons are critical for itch signaling (11, 12). In the spinal dorsal horn, estradiol replacement resulted in a significant increase in GRP messenger RNA (mRNA) compared with OVX + b group (Fig. 2*A*: one-way ANOVA post hoc test: *P < 0.05, b versus E; **P < 0.01; b versus EP, P versus EP) as well as an increase in GRP protein expression (Fig. 2*B*: one-way ANOVA post hoc test: **P < 0.01; b versus EP, P versus EP; Welch’s *t* test: *P < 0.05, E versus P). The dorsal horn expression of GRPR mRNA (Fig. 2*C*) or GRPR protein (Fig. 2*D*) was not affected by estradiol replacement. There was no significant effect of estradiol or progesterone replacement on the expression of the mRNA for the histamine H1 receptor (Fig. 2*E*), the neurokinin-1 receptor (NK1R) (Fig. 2*F*) involved in spinal nociceptive transmission (16), or for Bhlhb5 expressed in spinal BI-5 itch-inhibitory interneurons (Fig. 2*G*) (17).

**Fig. 3.**
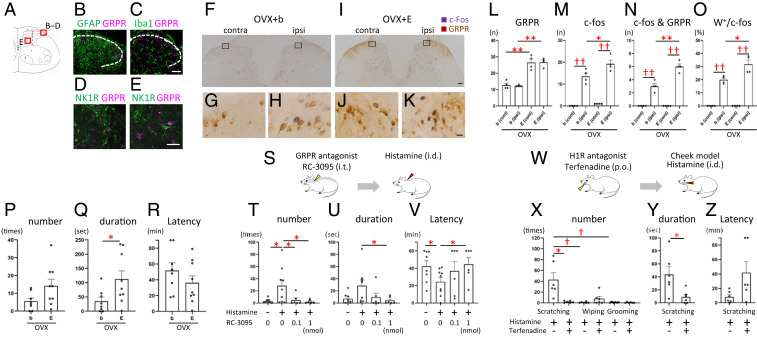
Estrogens increase the histamine-induced activation of spinal GRPR neurons and itch behavior. (*A*) Schematic showing the laminar organization of the rat L4 spinal cord. (*B*–*E*) Double-fluorescence images showing GRPR and other markers. Double-labeling of GRPR (magenta) and GFAP (green) (*B*), Iba1 (green) (*C*), and NK1R (green) in the superficial layers (*D*) and inner layers (*E*). (*F*–*K*) Representative double immunostaining of histamine-induced c-Fos (purple) and GRPR neurons (brown) in OVX + b and OVX + E rats. Boxed areas of *F* and *I* are enlarged in *G* and *H* and *J* and *K,* respectively. Histamine was injected unilaterally into the paw. *G* and *J*: control side; *H* and *K*: histamine injection side. (*L*–*O*) Mean number of GRPR (*L*), c-Fos (*M*), c-Fos/GRPR double-positive neurons (*N*), and percentage of c-Fos positive neurons that were double-labeled for GRPR (*O*) of OVX + b and OVX + E groups in the superficial layers of the spinal dorsal horn after unilateral injection of histamine. *n* = 4 rats/group. (*L*) Estradiol treatment increased the number of GRPR-expressing neurons compared with the OVX + b group [contralateral side: Student’s *t* test: T (6) = 5.840, *P* = 0.001; ipsilateral side: Student’s *t* test: T (6) = 10.610, *P* = 0.000]. (*M*) Histamine-induced c-Fos expression was increased on the ipsilateral side of the OVX + b [Welch’s *t* test: T (3.027) = 8.541, *P* = 0.003] and OVX + E group [Student’s *t* test: T (6) = 10.724, *P* = 0.000]. C-Fos expression on the ipsilateral side was higher in the OVX + E compared with the OVX + b group [Student’s *t* test: T (6) = 2.520, *P* = 0.045]. (*N*) c-Fos and GRPR double-positive neurons were increased in the ipsilateral side of the OVX + b [Student’s *t* test: T (6) = 6.273, *P* = 0.001] and OVX + E group [Student’s *t* test: T (6) = 14.697, *P* = 0.000]. Double positive neurons of the ipsilateral side were higher in the OVX + E compared with the OVX + b group [Student’s *t* test: T (6) = 5.542, *P* = 0.001]. (*O*) The proportion of both c-Fos and GRPR positive nerves in c-Fos–expressing neurons was increased in the ipsilateral side of the OVX + b [Welch’s *t* test: T (3) = 14.391, *P* = 0.001] and OVX + E group [Welch’s *t* test: T (3) = 9.546, *P* = 0.002]. This proportion of the ipsilateral side was higher in the OVX + E compared with the OVX + b group [Welch’s *t* test: T (4.006) = 3.294, *P* = 0.030). *P < 0.05, **P < 0.01; OVX + b versus OVX + E, ^††^*P* < 0.01; contralateral (cont) versus ipsilateral (ipsi) side of the spinal cord in each group. (*P*–*R*) Histamine-induced biting behavior of the hind paw. *n* = 8 and 9 rats, respectively. (*P*) The number of histamine-induced bites was increased in the estrogen treatment group [Student’s *t* test: T (15) = 2.012, *P* = 0.063]. (*Q*) The duration of histamine-induced bites was markedly increased in the estrogen treatment group compared with control [Welch’s *t* test: T (11.402) = 2.368, *P* = 0.037]. (*R*) There was no difference in histamine-evoked biting latency between the two groups [Student’s *t* test: T (15) = 1.178, *P* = 0.257]. *P < 0.05. (*S*–*V*) The effect of intrathecal injection of the GRPR antagonists on peripheral histamine-induced scratching behavior. Intrathecal injection is either RC-3095 or vehicle (aCSF), and intradermal injection is either histamine or vehicle (saline). *n* = 6 to 9/group. (*T*) Intrathecal injection of GRPR antagonist (RC-3095) reduced scratching number elicited by intradermal injection of histamine (Mann–Whitney *U* test: U (15) = 15.500, Z = 2.000, *P* = 0.046, control (aCSF + saline) versus histamine with aCSF ; Mann–Whitney *U* test: U (13) = 8.000, Z = 2.255, *P* = 0.026, histamine with aCSF versus histamine with 0.1 nmol RC3095; Mann–Whitney *U* test: U (13) = 7.000, Z = 2.374, *P* = 0.018, histamine with aCSF versus histamine with 1 nmol RC3095). (*U*) Intrathecal injection of GRPR antagonist dose-dependently reduced scratching duration [Welch’s *t* test: T (9.569) = 2.158, *P* = 0.058, control versus histamine with aCSF; Mann–Whitney *U* test: U (13) = 13.000, Z = 1.656, *P* = 0.113, histamine with aCSF versus histamine with 0.1 nmol RC3095; Mann–Whitney *U* test: U (13) = 8.000, Z = 2.247, *P* = 0.026, histamine with aCSF versus histamine with 1 nmol RC3095]. (*V*) Intrathecal injection of GRPR antagonist showed dose-dependently longer latency to scratch [Student’s *t* test: T (15) = 2.261, *P* = 0.039, control versus histamine with aCSF; Mann–Whitney *U* test: U (13) = 20.500, Z = 0.769, *P* = 0.456, histamine with aCSF versus histamine with 0.1 nmol RC3095; Student’s *t* test: T (13) = 2.340, *P* = 0.036, histamine with aCSF versus histamine with 1 nmol RC3095]. *P < 0.05. (*W*–*Z*) Effects of the histamine H1 receptor antagonist terfenadine on the histamine-induced itch. *n* = 6/group. (*X*) Intradermal injection of histamine into the cheek evoked primarily hindlimb scratching behavior, a marker of itch, and very little pain-related wiping or grooming behavior in the estrogen treatment group [Kruskal–Wallis test: *H* = 12.190, df = 2, *P* = 0.002; Bonferroni-corrected Mann–Whitney *U* post hoc test: U (10) = 0.000, Z = 2.892, *P* = 0.002, scratching versus wiping; U (10) = 0.000, Z = 2.908, *P* = 0.002, scratching versus grooming; U (10) = 11.500, Z = 1.092, *P* = 0.310, wiping versus grooming]. The H1R antagonist terfenadine significantly reduced histamine-evoked scratching numbers [Mann–Whitney *U* test: U (10) = 4.000, Z = 2.246, *P* = 0.026]. (*Y*) The H1R antagonist significantly reduced histamine-evoked scratching duration [Student’s *t* test: T (10) = 2.661, *P* = 0.024]. (*Z*) The H1R antagonist tended to have longer latency but not significant difference [Welch’s *t* test: T (5.428) = 2.073, *P* = 0.089]. ^†^*P* < 0.016 among histamine-evoked scratching, wiping, and grooming behavior. *P < 0.05, histamine versus histamine with terfenadine. Data are mean ± SEM. NK1R, neurokinin 1 receptor; GRPR, gastrin-releasing peptide receptor; C, contralateral side; I, ipsilateral side; OVX, ovariectomy; b, blank; E, estradiol treatment group; i.t., intrathecal injection; i.d., intradermal injection; aCSF, artificial cerebrospinal fluid; p.o., oral administration; H1R, Histamine H1 receptor. [Scale bars, 50 μm (*B*–*E*); 100 μm (*F* and *I*); and 10 μm (*G*, *H* and *J*, *K*).] See also *SI Appendix*, Fig. S4.

We then investigated whether estrogen-dependent histaminergic itch involves the functional activation of GRPR-expressing neurons in the spinal cord. These GRPR neurons were localized in the spinal dorsal horn and caudal part of the spinal trigeminal nucleus, crucial relay points for itch transmission (*SI Appendix*, Fig. S4). In GRPR-mRFP1 transgenic rats, GRPR expression was neither observed in glial cells (i.e., astrocytes and microglia) (Fig. 3 *A*–*C*) nor coexpressed in NK1R-expressing neurons (Fig. 3 *D* and *E*) in the superficial layers of the spinal cord (*SI Appendix*, Fig. S4). Intradermal injection of histamine in the dorsal hind paw of the OVX + E treatment group resulted in a significant increase in the number of spinal dorsal horn neurons positive for c-Fos, a marker for neuronal activation (Fig. 3 *F*–*K* and *M*: Welch’s *t* test: ^††^*P* < 0.01, contralateral (C) versus ipsilateral (I) side of OVX + b; Student’s *t* test: ^††^*P* < 0.01, C versus I side of OVX + E; Student’s *t* test: *P < 0.05, OVX + b versus OVX + E in the I side), c-Fos/GRPR double-positive neurons (Fig. 3*N*: Student’s *t* test: ^††^*P* < 0.01, C versus I side of OVX + b; Student’s *t* test: ^††^*P* < 0.01, C versus I side of OVX + E; Student’s *t* test: **P < 0.01, OVX + b versus OVX + E in the I side), and percentages (Fig. 3*O*: Welch’s *t* test: ^††^*P* < 0.01, C versus I side of OVX + b; Welch’s *t* test: ^††^*P* < 0.01, C versus I side of OVX + E; Welch’s *t* test: *P < 0.05, OVX + b versus OVX + E in the I side) in ipsilateral superficial laminae of the dorsal horn compared with the OVX + b group. Estradiol replacement by itself resulted in a marked increase in the number of GRPR-positive neurons (Fig. 3*L*: Student’s *t* test: **P < 0.01, OVX + b versus OVX + E in the C side; Student’s *t* test: **P < 0.01, OVX + b versus OVX + E in the I side). In addition, intradermal injection of histamine in the hind paw of the OVX + E group resulted in a significant increase in hind paw biting behavior, which is thought to reflect itch (18) (Fig. 3 *P* and *Q*: biting number: Student’s *t* test: *P* = 0.063; biting duration: Welch’s *t* test: *P < 0.05, OVX + b versus OVX + E) with no change in latency to hind paw biting (Fig. 3*R*). Furthermore, intrathecal administration of the GRPR antagonist RC3095 dose-dependently reduced histamine-evoked scratching behavior in the estrogen-treated females (Fig. 3 *S*–*V*: scratching number: Mann–Whitney *U* test: *P < 0.05, control [aCSF + saline] versus histamine with aCSF, histamine with aCSF versus histamine with 0.1 nmol RC3095, histamine with aCSF versus histamine with 1 nmol RC3095; scratching duration: Welch’s *t* test: *P* = 0.058, control versus histamine with aCSF; Mann–Whitney *U* test: *P < 0.05, histamine with aCSF versus histamine with 1 nmol RC3095; latency: Student’s *t* test: *P < 0.05, control versus histamine with aCSF, histamine with aCSF versus histamine with 1 nmol RC3095). Using the “cheek” model (19) (Fig. 3*W*), histamine elicited hind paw scratch bouts indicative of itch in the estrogen-treated group but very few ipsilateral forelimb wipes indicative of pain or grooming behavior (Fig. 3*X*: Kruskal–Wallis post hoc test: ^†^*P* < 0.016, scratching versus wiping, scratching versus grooming). Such scratching was significantly attenuated by the histamine H1 receptor antagonist terfenadine (Fig. 3 *X* and *Y*: scratch bouts: Mann–Whitney *U* test: *P < 0.05; scratch duration: Student’s *t* test: *P < 0.05, histamine versus histamine with terfenadine), and latency to scratching tended to increase following terfenadine (Fig. 3*Z*: Welch’s *t* test: *P* = 0.089). These data indicate that histamine-evoked itch is mediated by peripheral histamine H1 receptor and spinal GRPR-expressing neurons and that estrogens could enhance their activity.

**Fig. 4.**
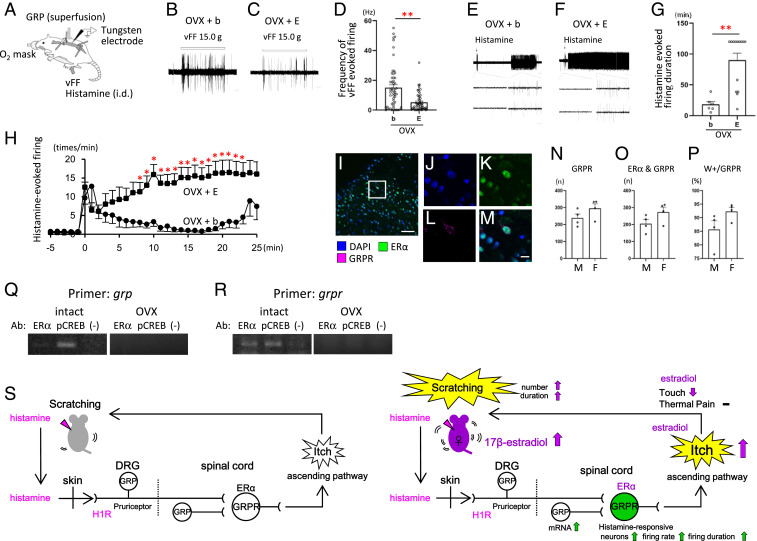
Estrogens increase spinal GRPR neuronal activity evoked by histamine. (*A*) Schematic illustration of in vivo extracellular recordings from the rat spinal dorsal horn. (*B* and *C*) Representative traces of von Frey filament (vFF)-evoked firing of neurons recorded in OVX + b (*B*) and OVX + E (*C*) rats. (*D*) Frequency of vFF-evoked firing was reduced in the OVX + E compared with OVX + b group [*n* = 54 and 55 neurons/group, respectively, Mann–Whitney *U* test: U (107) = 866.000, Z = 3.753, *P* = 0.000]. (*E* and *F*) Representative traces of the responses elicited by intradermal injection of histamine in GRP-sensitive neurons recorded in OVX + b (*E*) and OVX + E (*F*) treated rats. (*G*) Histamine-evoked firing of the GRP-responsive neurons persisted for a significantly longer time in OVX + E compared with OVX + b group [*n* = 6 and 14 neurons, respectively, Mann–Whitney *U* test: U (18) = 8.000, Z = 2.940, *P* = 0.003]. (*H*) Graph plots of the mean firing rate (impulses/min) of neurons recorded in OVX + E (black filled squares) (*n* = 14) and OVX + b (black filled circles) (*n* = 6) groups. Estrogens increased the histamine-evoked firing rate in the OVX + E compared with OVX + b group (Mann–Whitney *U* test). (*I*–*M*) Representative fluorescent image of DAPI- (blue: *I*, *J*, and *M*), ER-α– (green: *I*, *K*, and *M*), and RFP-labeled GRPR-expressing neurons (magenta: *I*, *L*, and *M*) in the spinal dorsal horn. Boxed areas of *I* is enlarged in *J* through *M*. [Scale bars, 50 μm (*I*); 10 μm (*J*–*M*).] (*N*–*P*) Expression and colocalization of the GRPR and ER-α in the superficial layers of the cervical spinal cord. (*N*) The number of the GRPR-expressing cells labeled with RFP in male and proestrus female rats [*n* = 4 rats/group, Student’s *t* test: T (6) = 1.562, *P* = 0.169, M versus F]. (*O*) The number of the ER-α and GRPR double-positive cells in male and female rats [Student’s *t* test: T (6) = 1.818, *P* = 0.119, M versus F]. (*P*) Percentage of the ER-α and GRPR double-positive cells in the GRPR-expressing cells in male and female rats [Student’s *t* test: T (6) = 1.903, *P* = 0.106, M versus F]. (*Q* and *R*) Immunoprecipitated chromatin containing ER-α or pCREB. *Grp* promotor (*Q*) and *grpr* promotor (*R*) DNA in the precipitate was analyzed by PCR. ChIP was conducted with intact females (*Left*) and OVX females (*Right*). Each panel shows ER-α (*Left Lane*), pCREB (*Middle Lane*), and control omitting the antibody (−) (*Right Lane*) to confirm the specificity of immunoprecipitation. (*S*) Schematic diagram illustrating estradiol enhancement of histamine-evoked itch via the spinal GRP/GRPR system (45, 46). *P < 0.05, **P < 0.01; OVX + b versus OVX + E. Data are mean ± SEM. vFF, von Frey filament; i.d., intradermal injection; OVX, ovariectomy; b, blank; E, estradiol treatment group. [Scale bars, 1 s (*B* and *C*); 2 min (*Upper* part of *E*); 5 min (*Upper* part of *F*); 100 msec (*Lower* parts of *E* and *F*).] See also *SI Appendix*, Table S1 and Figs. S5–S7.

Next, we analyzed itch-related neural activity using in vivo extracellular recordings from neurons in superficial laminae I and II (<100 µm from the surface; *SI Appendix*, Fig. S5) of the rat spinal dorsal horn (Fig. 4*A*). Spontaneous firing of dorsal horn neurons was relatively low in the OVX + b or OVX + E group (*SI Appendix*, Fig. S6). Neuronal responses elicited by an innocuous von Frey filament were significantly lower in the OVX + E compared with the OVX + b group (Fig. 4 *B*–*D*: Mann–Whitney *U* test: **P < 0.01, OVX + b versus OVX + E), consistent with the behavioral data (Fig. 1*H*). Subsequently, GRP was superfused to the surface of the spinal cord to search for responsive neurons. A total of 25% of neurons (14/55) in the OVX + b group and 30% (16/54) of neurons in the OVX + E group responded to GRP (*SI Appendix*, Fig. S7). These GRP-responsive neurons were then tested for responsiveness to intradermal hind paw injection of histamine. A total of 31% of neurons in the OVX + b group responded to histamine, while a significantly higher proportion, 75%, in the OVX + E group responded to histamine (χ^2^ test: *P* < 0.01) (*SI Appendix*, Table S1). The duration of histamine-evoked firing was significantly longer in the OVX + E group than in the OVX + b group (Fig. 4 *E*–*H*: Mann–Whitney *U* test: **P < 0.01; Mann–Whitney *U* test: *P < 0.05, OVX + b versus OVX + E). These results demonstrate that estrogen treatment increased the frequency and duration of histamine-evoked activity of spinal GRPR-expressing neurons, consistent with the behavioral data showing that estradiol increased the number of histamine-evoked scratch bouts and overall scratching duration (Fig. 1 *B*, *C*, *E*, and *F* and *SI Appendix*, Fig. S1).

Finally, to understand the mechanism by which estrogens regulate itch transmission through GRP and GRPR, we focused on the nuclear estrogen receptor α (ERα), which is a transcription factor. After ligand binding, the nuclear receptors bind directly to the specific sequence of DNA on the target gene promotor known as the hormone responsive element to regulate transcription. We found the estrogen responsive element (ERE) on the promotor region of the genes for both GRP and GRPR. At first, to know the interaction of ER-α and GRPR, we analyzed the colocalization of ERα- and GRPR-positive neurons in the spinal dorsal horn. We found that many GRPR-expressing neurons coexpressed ER-α in the superficial layers of the spinal dorsal horn (Fig. 4 *I*–*P*: 85.7 ± 3.2% colocalization in male and 92.3 ± 1.5% in females). Next, the chromatin immunoprecipitation (ChIP) assay was used to examine whether the transcription factor ER-α binds to the GRP or GRPR promoter region and also to examine cAMP response element binding protein (CREB)-mediated transcription, because estrogens and ER-α have a CREB-mediated transcriptional pathway (20–22). In the spinal dorsal horn immunoprecipitation assay, we found that pCREB was bound to the GRP promoter (Fig. 4 *Q*, *Left*) and ER-α and pCREB were bound to the GRPR promoter (Fig. 4 *R*, *Left*) in intact females. However, we did not observe ER-α or pCREB to bind to either the GRP or GRPR promotor in the OVX females (Fig. 4 *Q* and *R*, *Right*). These results suggest that estrogens regulate the transcription of GRPR, which is essential for itch transmission, via ER-α and CREB, and that CREB is involved in the transcriptional regulation of GRP. Thus, estrogens selectively regulate histamine-evoked itch in females via the spinal GRP/GRPR system (Fig. 4*S*).

## Discussion

The mechanisms of female-specific pruritus (4, 5, 7, 23) and sex differences in itch sensitivity (24–26) have long been elusive. Here, we show that estradiol, but not progesterone, likely enhances histamine-evoked itch in female rats via spinal GRPR-expressing neurons.

Several lines of evidence indicate that estrogens regulate histamine-evoked itch via activity of GRPR-expressing neurons in the spinal dorsal horn of female rats. First, physiological concentrations of estradiol replacement in ovariectomized female rats enhanced and sustained histamine-evoked scratching directed to the nape of the neck as well as biting behaviors directed to the hind paw. This enhancement was mediated via histamine H1 receptors and spinal GRPR-expressing neurons because an H1 receptor antagonist and a GRPR blocker inhibited the scratching in OVX + E rats. Second, estradiol replacement increased the histamine-evoked neural activity as assessed by c-Fos expression in spinal GRPR-expressing neurons. Third, electrophysiological data show that estrogens markedly increased the frequency and duration of histamine-evoked responses of spinal dorsal horn neurons without affecting spontaneous neural firing. Overall, our results directly demonstrate that the spinal GRP/GRPR system is specifically involved in female estrogen-dependent itch. In our study, physiological levels of estradiol treatment did not affect the threshold of thermal pain but decreased touch sensitivity and increased itch sensitivity via the itch-specific spinal GRP/GRPR system. Our results indicate that estradiol acts separately on the neural circuits that underlie these distinct somatosensory submodalities.

Estradiol not only increased itch sensitivity and activity of GRPR-expressing neurons but also the expression of GRP mRNA and the number of GRPR-positive neurons in the spinal dorsal horn of female rats. In contrast, progesterone had no effect on itch sensitivity or GRP expression. Treatment with both estradiol and progesterone resulted in increased GRP expression but not itch behavior, suggesting that progesterone inhibits the effects of estradiol on itch transmission in females. In the present study, we found a high degree of colocalization of ER-α and GRPR in the spinal dorsal horn. We also showed that estradiol affects the transcription of GRP and GRPR in the spinal dorsal horn via the transcription factor, ER-α, binding to ERE and CRE (cAMP response element) on the GRP and GRPR promotor regions. Because the CRE site was reported to be necessary for transcriptional activity of the GRPR promotor (27–29), estrogens can interact with CREB through ER-α to regulate transcription of several genes (20–22). It has been recently reported that an ERα-positive subpopulation of interneurons is involved in itch transmission in the spinal dorsal horn (30) and that activation of ER-α is associated with inflammatory pruritus in mice (31). Thus, our data indicates that ER-α is involved in the transmission of itch, and we have newly clarified that estrogens modulate itch sensitivity by regulating spinal GRP and GRPR gene transcription.

Histamine is the primary peripheral itch mediator in humans but elicits little if any itch-related scratching behavior in rats (24, 32); instead, serotonin elicits robust scratching in this species (32–34). This was confirmed by the present results in naïve male and female rats. On the other hand, our present study showed that, in the presence of estrogens, there was an enhancement of histamine-evoked itch sensitivity in female rats. Therefore, this rat model can be a useful for elucidating the mechanism of histamine-dependent itch. Spinal GRPR neurons are reported to be required for histaminergic and nonhistaminergic itch in male mice (11, 35, 36). Our study additionally supports a role for spinal GRPR-expressing neurons in histaminergic itch transmission in female rats. Previous studies indicated that spinal neurons that express the neuromedin B receptor (NMBR) are required for itch, including histaminergic itch. NMBR-expressing neurons function upstream of GRPR-expressing neurons in the spinal dorsal horn (37). This presynaptic or neuromedin B (NMB)/NMBR-mediated mechanism might play an important role, since histamine activated a higher proportion and longer duration of firing of GRP-responsive neurons in the OVX + E group than in OVX + b group, despite the observation that comparable proportions of neurons in these two groups responded to exogenous superfusion of GRP (25% in the OVX + b and 30% in the OVX + E group). The increased sensitivity to histamine-induced itch in the estrogen-treated group may be due to the transmission of itch via NMBR-expressing neurons. In addition, like histamine, estradiol enhanced itch sensitivity elicited by CQ, which also exhibited a female-dominant sex difference.

A recent study demonstrated that GRP in sensory neurons is required for CQ- but not histamine-evoked itch behavior using GRP^cre-KI^ mice and that spinal GRP neurons are dispensable for itch transmission (38). In contrast, our results showed that estrogen treatment increased *GRP* mRNA in the spinal cord, GRPR neural activity in the spinal dorsal horn, and itch behavior induced by histamine and CQ in female rats. Furthermore, estrogen treatment affected the transcription of GRP and GRPR in the spinal dorsal horn via ER-α and CREB, suggesting that responses of GRPR neurons to GRP are increased, with estrogens playing a regulatory role. In addition, genetic and pharmacological approaches provide evidence supporting the role of spinal GRP neurons in itch. Chemogenetic activation of GRP neurons in the spinal cord increased the CQ- and histamine-induced itch-related behavior but did not change the pain response (15). Chemogenetic inhibition of GRP neurons in the spinal cord suppressed both histaminergic and nonhistaminergic itch without affecting the mechanical pain threshold (39).

ER-α has been demonstrated to be expressed in small-diameter neurons of rat dorsal root ganglia (DRG), and its expression is regulated by circulating levels of 17β-estradiol (40). Therefore, it is possible that estrogens may additionally regulate GRP and NMB expression in the DRG via ER-α to increase scratching behavior induced by histamine and CQ (Fig. 4*S*).

In addition to pruritus during the period of pregnancy, there is an increase in the prevalence of atopic dermatitis in female patients at the onset of adolescence, when the blood estrogen concentration rapidly increases (4, 7, 41). Certain itchy skin diseases and allergic conjunctivitis with symptoms of itching show a greater prevalence in females than males (42, 43). Our findings may be related to these female-specific itchy conditions, but our study has certain limitations. First, the study was performed using an animal model, and there is no supporting evidence that a similar mechanism pertains to humans. Secondly, we used hormone replacement as a surrogate for pregnancy and did not investigate other changes during gestation that might contribute to itch. Third, while our data provides correlative evidence for the effects of estradiol on spinal GRP signaling, we did not currently perform loss-of-function experiments that are needed to demonstrate causality of the proposed mechanisms; these should be done in future studies. Taken together, we hope that our research will serve as the basis for the treatment of pruritus in women and a bridge to gender medicine.

## Materials and Methods

All experimental procedures were approved in accordance with the *Guide for the Care and Use of Laboratory Animals* (44) prepared by Okayama University (Okayama, Japan), by Kyoto Prefectural University of Medicine (Kyoto, Japan), by Toyama University (Toyama, Japan), and by the National Institute of Genetics (Shizuoka, Japan) and performed in accordance with the NIH guidelines on animal care. Adult wild-type Wistar rats and GRPR-mRFP transgenic rats were used in this study. To examine the effects of female sex steroid hormones, ovariectomized females were implanted with a blank capsule, a capsule containing physiological levels of 17β-estradiol, a capsule containing physiological levels of progesterone, or a capsule containing physiological levels of both hormones, for 1 to 2 mo. For itch behavioral analysis, rats received either saline, 3% histamine, or 10% CQ diphosphate salt diluted in saline via intradermal injection in the nape of the neck, cheek, or hind paw. Immediately after the injection, the rat was placed into the arena and videotaped from above for 60 min for scratching behavior. Mechanical sensitivity was assessed by the von Frey filament test, and thermal pain sensitivity was assessed by the Hargreaves test. For RT-PCR, enzyme-linked immunosorbent assay, Western blot, and the ChIP assay, the dorsal horns of the cervical spinal cords were collected and used for analysis. Complete methods are described in *SI Appendix*. Brain and spinal cord sections were used for immunofluorescence and immunoperoxidase histochemistry after perfusion with physiological saline followed by 4% paraformaldehyde in 0.1 M phosphate buffer. Antibody information is provided in *SI Appendix*, Fig. S2. In vivo extracellular single-unit recordings of superficial spinal dorsal horn (lamina I and II) neurons were performed in female rats. Statistical analyses were performed using SPSS Statistics version 27 (IBM). Graphs were made using GraphPad Prism 8 (GraphPad Software). Statistical analyses for each study are indicated in the figure legends. More detailed information on materials and methods is provided in *SI Appendix*.

## Supplementary Material

Supplementary File

## Data Availability

All study data are included in the article and/or *SI Appendix*.
