# Supplementary material for: Estrogens influence female itch sensitivity via the spinal gastrin-releasing peptide receptor neurons
Source: Proc Natl Acad Sci U S A. 2021 Jul 26;118(31):e2103536118. doi: 10.1073/pnas.2103536118 (PMC8346901; doi:10.1073/pnas.2103536118)
Supplement: Supplementary File [file pnas.2103536118.sapp.pdf]

Supplementary Information for

Estrogens influence female itch sensitivity *via* the spinal gastrin-releasing peptide receptor neurons

Keiko Takanami*, Daisuke Uta, Ken Ichi Matsuda, Mitsuhiro Kawata, Earl Carstens, Tatsuya Sakamoto, and Hirotaka Sakamoto

*Corresponding author. Email: takanami@nig.ac.jp

This PDF file includes:

Materials and Methods
Supplementary Table 1 to 2
Supplementary Figures S1 to S7
References (1–14)

Materials and methods

Animals

Adult wild-type Wistar rats (Shimizu Laboratory Supplies Co., Ltd., Kyoto, Japan or Charles River Japan, Yokohama, Japan) between 9 and 20 weeks old were used in this study. For experiments probing the expression site of the gastrin-releasing peptide receptor (GRPR), adult transgenic Wistar rats bred in the animal facilities of Okayama University were examined. We generated monomeric red fluorescent protein 1 (mRFP1)-human heparin-binding epidermal growth factor (human diphtheria toxin receptor: *Dxtr*) expressing BAC transgenic rats with the GRPR promoter driving fusion gene expression by pronuclear injection of Wistar rat embryos (Institute of Immunology Co., Ltd., Tokyo, Japan). This reproduced the endogenous *Grpr* pattern in the brain and spinal cord revealed by mRFP1 fluorescence. Using GRPR-mRFP1 transgenic rats, we found that GRPR-expressing cells labeled with RFP were localized in the brain and spinal cord as reported (1, 2). All rats were maintained on a 12-h light/12-h dark cycle and were provided unlimited access to water and rodent chow. All experimental procedures were approved in accordance with the Guide for the Care and Use of Laboratory Animals prepared by Okayama University (Okayama, Japan), by Kyoto Prefectural University of Medicine (Kyoto, Japan), by Toyama University (Toyama, Japan), and by National Institute of Genetics (Shizuoka, Japan) and performed in accordance with the National Institutes of Health guidelines on animal care. All efforts were made to minimize animal suffering and reduce the number of animals used in this study.

Hormone treatments

Adult wild-type Wistar rats were divided into 6 groups: sham-operated males, sham-operated females, bilaterally ovariectomized (OVX) females implanted with a

blank (b) capsule as a control (OVX + b), OVX females treated with 17β-estradiol (OVX + E), OVX females treated with progesterone (OVX + P), and OVX females treated with both estradiol and progesterone (OVX + EP). Adult female GRPR-mRFP1 transgenic rats were divided into 2 groups: OVX + b, and OVX + E. OVX, sham operations, and implantation were performed under deep anesthesia with isoflurane inhalation. All rats were implanted s.c. with 15-mm Silastic capsules (Dow Corning, inner diameter 1.57 mm; outer diameter 3.18 mm; Futuremedics Australia, Victoria, Australia) containing crystalline 17β-estradiol (Sigma, St. Louis, MO, USA), or with 40-mm Silastic capsules containing crystalline progesterone (Sigma) or a blank control capsule. Silastic capsules were cut to the required length plus an additional 10 mm. Each end of the tube was sealed with 5 mm of silicon sealant. Blood concentrations of estradiol and progesterone released constantly from these tubes were within the normal range of the estrus cycle in adult female rats from the previous papers (3, 4). Our data from the enzyme-linked immunosorbent assay showed the physiological concentration of estradiol in OVX + E group (Male: 12.06 ± 4.13, Female: 30.60 ± 6.46, OVX + b: 17.26 ± 3.43, OVX + E: 60.18 ± 13.54, OVX + P: 16.86 ± 2.50 pg/ml, n = 5, 6, 6, 6, 6, respectively). One–two months after surgery, rats were used for behavioral analysis, quantitative analysis for qPCR, western blot, immunohistochemistry, or chromatin immunoprecipitation assay.

Behavioral Analysis

Itch behavior

The fur on the rostral back was shaved and rats were habituated to the glass recording arena (30 cm long, 20 cm wide, 36 cm high) one week prior to testing. Six groups of adult rats (male, female, OVX + b, OVX + E, OVX + P, OVX + EP) received either saline, 3% histamine (H7125, Sigma), or 10% chloroquine diphosphate salt (C6628,

Sigma) diluted in saline *via* intradermal (id) injection in the nape of the neck (100 μl; *n* = 9–12 for each group) using a microsyringe. Two group of rats (OVX + b and OVX + E) received 3% histamine diluted in saline *via* id injection of the dorsal hindpaw (50 μl; *n* = 8–9 for each group) using a microsyringe. For intrathecal (it) injections, either vehicle (aCSF) or the GRP receptor antagonist RC-3095 (0.1–1 nmol; R9635; Sigma-Aldrich, St. Louis MO, USA) (*n* = 6–9 for each group) was administered (10 μl) by insertion of the polyurethane canula (MRE-025 36FT, inner diameter 0.30 mm; outer diameter 0.64 mm; Bio Research Center, Nagoya, Japan) into the cisterna magna and advancing the tip into the cervical enlargement of the spinal cord (2 cm length from C1 to C5–6 for 250–300g rats), followed 15 min later by id injection of 3% histamine (100 μl) or saline as a vehicle into the nape of the neck. For the cheek model of itch, fur on the cheek was shaved. Rats received an id injection of 3% histamine diluted in saline in the cheek (50 μl) using a microsyringe. Terfenadine (T9652, Sigma) , an H1R antagonist, was dissolved in tap water containing 0.5% sodium carboxymethyl cellulose (Wako Pure Chemical Industries, Osaka, Japan) and administered orally at a dose of 30 mg/kg, 30 min before 3% histamine id injection into cheek as described above (*n* = 6 / group) (5).

Immediately after the id injection the rat was placed into the arena and videotaped from above for 60 min for scratching behavior of nape of the neck and 90min for biting behavior of the paw and cheek model. Immediately after commencing videotaping all investigators left the room. Videotapes were reviewed by investigators blinded to the treatment, and the number of scratch bouts was counted at 1-min intervals. A scratch bout was defined as one or more rapid hindpaw motions directed toward and contacting the injection site, and ending with licking or biting of the toes and/or placement of the hindpaw on the floor.

Pain behavior

In each of the pain tests, 6 groups of adult rats (male, female, OVX + b, OVX + E, OVX + P, OVX + EP) (*n* = 8/group) were previously habituated to the behavioral apparatus for 20 min. The threshold for paw withdrawal elicited by innocuous von Frey filaments was determined as previously described (6). The nylon von Frey filament was pressed against the planter of the rat from beneath the net, and it was noted if the animal raised its hindpaw in an escape response. If two escape behaviors were observed before 10 stimuli were applied, the experiment was terminated.

Thermal pain sensitivity was assessed in the same rats using the Hargreaves test. The rat was placed on a clear glass surface warmed to 30°C, and the latency for paw withdrawal elicited by a radiant heat stimulus delivered to the plantar surface was measured using a PAW Thermal Stimulator (UC San Diego). A 20-sec cutoff was imposed to prevent tissue damage. The mean of three paw withdrawal latency measurements taken at 5 min intervals as determined for each rat.

Real-time PCR

Six groups of adult rats (male, female, OVX + b, OVX + E, OVX + P, OVX + EP) (*n* = 5–8/group) were euthanized by decapitation under deep sodium pentobarbital anesthesia (50 mg/kg body weight). Cervical spinal cords (dorsal region) and cervical DRGs were quickly removed on ice, and frozen by dry ice. Dissected tissue was immediately fixed with Ambion RNA*Later* solution (Thermo Fisher Scientific, Waltham, MA) and stored at –30°C until RNA extraction. Total RNA was extracted using an illustra RNAspin Mini RNA isolation kit (GE Health Care, Buckingham, United Kingdom) according to the manufacturer's protocol. The concentration of total RNA was measured using a Qubit RNA assay kit (Thermo Fisher Scientific). First-strand cDNA was synthesized from 400 ng of total RNA with random primers

using an Omniscript RT kit (QIAGEN, Hilden, Germany). To determine the effect of female sex steroid hormones on the *Grp, Grpr, histamine H1 receptor (H1r), neurokinin 1 receptor (NK1R), Bhlhb5,* and *Gapdh* transcripts in the dorsal cervical spinal cords or cervical DRGs, 20 ng cDNAs from the cervical cord and DRG of 6 groups of adult Wistar rats (male, female, OVX + b, OVX + E, OVX + P, OVX + EP) were subjected to qPCR, which was performed in 20-μL reaction volumes consisting of Taqman Gene Expression Assays (Applied Biosystems, Foster, CA) by using a CFX96 qPCR detection system (Bio-Rad Laboratories). We used the TaqMan qPCR methodology for rat *Grp, Grpr, Hrh1(*H1r*), Tacr1* (NK1R)*, Bhlhe22* (Bhlhb5), and *Gapdh* (Taqman Gene Expression Assays no. Rn00592059, Rn01420745, Rn00566691-S1, Rn00562004, Rn01755778, and Rn99999916). Amplification was carried out at 95°C for 10 minutes and 50 cycles with 95°C for 10 seconds, 60°C for 30 seconds. The expression in each reaction was normalized by the expression of *Gapdh* as an internal control. Duplicate qPCR analysis was performed for each sample.

ELISA

Six groups of adult rats (male, female, OVX + b, OVX + E, OVX + P, OVX + EP) (*n* = 5–7/group) were euthanized by decapitation under deep sodium pentobarbital anesthesia (50 mg/kg body weight). Cervical spinal cords (dorsal region) were quickly removed on ice, weighed, snap frozen immediately in liquid nitrogen, and used for peptide extraction. Peptides were extracted according to our previous methods (7). Frozen tissues were homogenized in 5% acetic acid using a disposable homogenizer (BioMasher; Nippi, Tokyo, Japan) and boiled for 10 min. The homogenate was centrifuged at 15,000 × *g* for 10 min at 4°C. The supernatant was collected in a tube, and the precipitate was again homogenized and centrifuged. The two supernatants

were pooled and forced through a disposable C-18 cartridge (SPE, 1 ml to 100 mg; SILICYCLE, Quebec, Canada). The retained material was then eluted with 60% methanol. The elute was concentrated in a vacuum centrifuge and subjected to competitive ELISA specific for GRP using a kit for GRP (Phoenix Pharmaceuticals, Inc., Burlingame, CA) according to the manufacturer's protocol. The concentration of GRP was calculated in terms of picomoles per gram wet weight (picomoles per gram tissue) of each spinal cord. We included the standard curve in each experiment.

Western Blot

Western blotting was conducted according to our previously described methods (8). In brief, 6 groups of adult rats (male, female, OVX + b, OVX + E, OVX + P, OVX + EP) (*n* = 7–10/group) were sacrificed by blood loss under deep pentobarbital anesthesia. Cervical spinal cords were quickly removed and placed on dry ice, and spinal dorsal horns were carefully dissected. Samples were snap-frozen immediately in liquid nitrogen and used for Western analysis. Lysate samples for GRPR measurement (50 µg cervical spinal dorsal horn protein) were boiled in 10 µL sample buffer containing 62.5 mM trishydroxymethyl-aminomethane-HCl (Tris-HCl; pH. 6.8), 2% SDS, 25% glycerol, 10% 2-mercaptoethanol, and a small amount of bromophenol blue. Samples were then separated by 4%–20% gradient SDS-PAGE and electroblotted onto polyvinylidene difluoride (PVDF) membranes (Bio-Rad Laboratories, Hercules, CA, USA) using a semidry blotting apparatus (Bio-Rad Laboratories). Membranes were blocked with the PVDF Blocking Reagent for the Can Get Signal kit (TOYOBO, Tokyo, Japan) for 30 min at room temperature and then incubated overnight at 4ºC in Can Get Signal Solution 1 (TOYOBO) containing a 1:1,000 dilution of rabbit polyclonal antibody against human GRPR (GTX100015, GeneTex, Irvine, CA). Blotted membranes were washed three times with 0.05%

Tween 20 in Tris-HCl buffered saline (TBST) and incubated with horseradish peroxidase (HRP)-conjugated goat polyclonal antibody against rabbit IgG (Bio-Rad Laboratories) at a 1:10,000 dilution in Can Get Signal Solution 2 (TOYOBO) for 1 hr at room temperature. After washing five times with TBST, blots were visualized by the Immun-Star WesternC Chemiluminescence Kit (Bio-Rad Laboratories). Images of immunoblots were detected by ChemiDoc™ XRS+ System with Image Lab™ Software (Bio-Rad) and adjusted slightly for brightness and contrast to provide a uniform background.

Tissue preparation for immunohistochemistry

Adult GRPR-mRFP1 transgenic rats were deeply anesthetized by intraperitoneal injection of sodium pentobarbital (50 mg/kg body weight) and transcardially perfused with physiological saline followed by 4% paraformaldehyde in 0.1 M phosphate buffer (PB). For the c-Fos analysis, OVX + b and OVX + E female rats (n = 4 of each) were perfused using the same procedure 120 min after id injection of 3% Histamine to the right foot. For the ERα expression analysis in the GRPR neurons, male and proestrus female rats (n = 4 of each) were perfused. Vaginal smears were collected daily and the estrus cycle (proestrus, estrus, metestrus, and diestrus) was determined from the morphology of the nucleated epithelial cells, keratinocytes, and leukocytes. Brain and spinal cords were removed, immersed in the same fixative for 3 hr at room temperature, and then immersed in 25% sucrose in 0.1 M PB for 48 h at 4°C. Then, tissues were quickly frozen using powdered dry ice and cut into 30 µm-thick sections on a cryostat (CM3050 S; Leica, Nussloch, Germany). These sections were then washed several times (5 min/wash) with phosphate-buffered saline (PBS).

Immunofluorescence

We performed immunofluorescence analysis according to our established methods (9). The sections were incubated with the primary rabbit antiserum against rat $GRP_{20\text{-}29}$ (11081; 1:1,000 dilution; AssayPro, St. Charles, MO), a mouse monoclonal antibody against GFAP (G3893; 1:1,000 dilution; Sigma-Aldrich), a mouse monoclonal antibody against Iba1/AIF1 (MABN92; 1:1,000 dilution; Millipore, Bedford, MA), a primary rabbit antiserum against NK1R (S8305; 1:5,000 dilution; Sigma-Aldrich) , or a primary rabbit antiserum against ERα (C1355; 1:2,000 dilution, Millipore) for ~48 hours at 4°C after blocking nonspecific binding with 1% bovine serum albumin (BSA), 1% normal goat serum and 0.3% Triton X-100 in PBS for 1 hr at room temperature. The sections were incubated for 2hr at room temperature with Alexa Fluor 546-linked anti-mouse IgG raised in goats (Molecular Probes, Eugene, OR, USA) or Alexa Fluor 488-linked anti-rabbit IgG raised in goats (Molecular Probes) using at a 1:1,000 dilution for detection. For the ERα expression analysis, after the 2nd antibody reaction, the sections were incubated with DAPI (D9542; Sigma). Sections were imaged with a confocal laser scanning microscopy (FluoView 1000, Olympus, Tokyo, Japan) and an Olympus FSX100 fluorescent microscope (Olympus). Analysis of ERα expression in the GRPR-expressing neurons in the cervical spinal cord (C1 to C8 level) was performed using spinal cross-sections in male and female groups. Spinal cord tissue was cut in 30 µm thick sections and every 4th section was taken to avoid duplicate counts. Then, using 12 sections per animal, we counted the number of ERα immunoreactive-, RFP labeled-neurons in the superficial layers (I-II layers) of the spinal cord, as an indicator of GRPR expression and double-positive neurons expressing both ERα and RFP. First, the mean number of immunopositive cells in each animal was calculated, and then the average value in each group was calculated. The antibodies used in this study are shown in supplementary Table 2.

Immunoperoxidase histochemistry

Sections were first incubated with 1% H_2O_2 in absolute methanol for 20 min to eliminate endogenous peroxidase activity. Sections were then rinsed with PBS three times (5 min/rinse). After blocking nonspecific binding with 1% normal goat serum and 1% BSA in PBS containing 0.1% Triton X-100 for 1 hr at room temperature, sections were incubated with a primary rabbit antiserum against c-Fos (PC38; 1:10,000 dilution; Millipore) for 48 hr at 4°C. Immunolabeling was detected using a streptavidin-biotin kit (Nichirei, Tokyo, Japan) followed by diaminobenzidine development with $NiCl_2$ enhancement to label c-Fos expressing cells purple. Then, sections were incubated with 1% H_2O_2 in absolute methanol for 20 min and with 1% normal goat serum and 1% BSA in PBS containing 0.1% Triton X-100 for 1 hr at room temperature. Sections were incubated with a mouse monoclonal antibody against RFP (M155-3; 1:10,000 dilution; MBL Nagoya, Japan) for 48 hr at 4°C. Immunolabeling was detected using a streptavidin-biotin kit (Nichirei, Tokyo, Japan) followed by diaminobenzidine development to label RFP expressing cells brown. Immunoreacted sections were analyzed using an Olympus FSX microscope (Olympus). Images were captured by a CCD camera and saved in TIFF format. Digital photomicrographs were processed with Adobe Photoshop CS5.1 at 300 dpi resolution. Analysis of c-Fos expression in the GRPR-expressing neuron in the lumbar spinal cord (L1 to L6 level) 120 min after injection of histamine into right paw was performed using spinal cross-sections in OVX + b and OVX + E groups. Spinal cord tissue was cut in 30 µm thick sections and every 4th section was taken to avoid duplicate counts. We confirmed the expression of c-Fos in the lumbar spinal cord was mainly expressed at the L4 to L5 levels. Then, using 7–10 sections per animal of the superficial layers (I-II layers) of L4 to L5 spinal cord, we counted the number of c-Fos immunoreactive-,

RFP immunoreactive-neurons as an indicator of GRPR expression and double positive neurons expressing both c-Fos and RFP on the contralateral and ipsilateral sides. First, the mean number of immunopositive cells in one section per each animal was calculated, and then the average value in each group was calculated. C-Fos+ neurons were identified by the following characteristics: densely positive staining in the clear round nuclei. RFP+ neurons were identified by the following characteristics: densely positive staining of cytoplasm with clear round nuclei. All micrographs were coded and evaluated without the knowledge of the experimental group designation, and the code was not broken until the analysis was complete. The antibodies used in this study are shown in supplementary Table 2.

***In vivo* extracellular recording from superficial dorsal horn neurons**

The methods used for the *in vivo* extracellular recording from the superficial spinal dorsal horn were similar to those described previously (10, 11). Briefly, adult female Wistar rats were anesthetized with urethane (1.2–1.5 g/kg, i.p.). Urethane produces a long-lasting steady level of anesthesia which does not require administration of additional doses except in a few cases. A thoracolumbar laminectomy was performed exposing the spinal cord at L1–L6 levels and the animal was then placed in a stereotaxic apparatus. After removing the dura and cutting the arachnoid membrane to make a window large enough for insertion of a tungsten microelectrode, the surface of the spinal cord was irrigated with 95% O_2–5% CO_2-equilibrated Krebs solution (10–15 ml/min) containing the following (in mM): 117 NaCl, 3.6 KCl, 2.5 $CaCl_2$, 1.2 $MgCl_2$, 1.2 NaH_2PO_4, 11 glucose, and 25 $NaHCO_3$, through glass pipettes at 37 ± 1°C. Extracellular single-unit recordings of superficial spinal dorsal horn (lamina I and II) neurons were performed as described previously (10, 11). Recordings were obtained from superficial dorsal horn neurons at a depth of 20–150 μm from the surface.

These cells were within the superficial dorsal horn as assessed by slices obtained from the same spinal level of age-matched mice. Unit signals were acquired with an amplifier (EX1; Dagan corporation, Minneapolis, USA). The data were digitized with an analog-to-digital converter (Digidata 1400A, Molecular Devices, Union City, CA, USA), stored on a personal computer with a data acquisition program (Clampex version 10.2; Molecular Devices), and analyzed with a special software package (Clampfit version 10.2; Molecular Devices). We searched the area on the skin where touch (with a cotton wisp) or noxious pinch (with forceps) stimuli elicited a neural response. The mechanical stimuli were applied with a fine von Frey filament (vFF) having a bending force of 15.0 g to skin folds on the ipsilateral hindlimb at the center of the unit's receptive field. The vFF stimulus was applied for 10 seconds. Histamine or saline was injected into the receptive area id through a 30-gauge needle. Spinal superfusion of GRP (SP2770b, ABGENT-a WuXi AppTec Company), RC-3095 (R9653, Sigma-Aldrich), and CNQX (ALX-550-042-M010, Enzo Life Sciences) was performed as previously described (12, 13). The criterion for a response to GRP was that the neuron's firing rate increased by 30% or more after GRP superfusion compared with the same time interval prior to GRP superfusion, as verified by showing a significant difference in mean firing rates pre *vs* post-GRP using a *t*-test.

Chromatin immunoprecipitation (ChIP) assay

ChIP was performed using a Magna ChIP A/G kit (Millipore) as described previously (14). Adult intact female and OVX female rats (n = 6) were deeply anesthetized by intraperitoneal injection of sodium pentobarbital (50 mg/kg body weight) and transcardially perfused with physiological saline followed by 4% paraformaldehyde in 0.1 M PB. Fixed cervical spinal cords were cut at a thickness of 1 mm and tissue fragments of the dorsal horn gray matter were bilaterally isolated using a stainless steel

cannula (inner diameter, 0.65 mm). The spinal cord fragments (n = 12) were sonicated to shear the chromatin. The chromatin solution was immunoprecipitated using anti-ERα (C1355; Millipore), or anti-phosphor-CREB (06-519; Merck). Purified DNA fragments were subjected to PCR amplification. Primers used for detection of the *grp* and *grpr* promoter were as follows: *grp* promoter forward: GGTTCGTGATACCTCGTTGG; *grp* promoter reverse: AGCACACTGACGTCTCCCTA; *grpr* promoter forward: CGCTGTATATTCCTGGCTCC; and *grpr* promoter reverse: CTCACACAGCATTTCCCCGT. PCR products were separated on a 2% agarose gel.

Statistics

All variables were tested for distribution normality using the Shapiro-Wilk normality test. For between-group comparisons, the Levene test for the equality of variance was performed after the normality test. After confirming equal variance, the statistical difference was determined by unpaired two-sided Student's *t*-test. In the case of unequal variance, an unpaired two-sided Welch's t-test was performed. If the data did not follow a normal distribution, statistical difference was determined by an unpaired two-sided Mann Whitney-*U* test. For comparison of more than two groups, once normality was confirmed by the Shapiro-Wilk normality test, one-way analysis of variance (ANOVA) was used. If the ANOVA was significant, and if equal variance was confirmed by the Levene test, *post-hoc* pairwise comparisons were conducted using Tukey's test. In the event of unequal variance, *post-hoc* pairwise comparisons were conducted using Gomes-Howell's test. If the data did not follow a normal distribution, statistical difference was determined by the Kruskal-Wallis test. If the Kruskal-Wallis test was significant, *post-hoc* pairwise comparisons were conducted using the Bonferroni-corrected Mann-Whitney *U* *post-hoc* test. The Pearson's

correlation coefficient test was used for correlation analysis. Results are expressed as the mean ± standard error of the mean (SEM). Statistical analyses were performed using SPSS Statistics version 27 (IBM, Chicago, IL, USA). $P < 0.05$ was considered statistically significant. For the Bonferroni-corrected Mann-Whitney *U post hoc* analysis following the Kruskal-Wallis test, $P < 0.0083$ was considered statistically significant in the 4 group comparison (b *vs* E *vs* P *vs* EP group). Graphs were made using GraphPad Prism 8 (GraphPad Software, San Diego, CA, USA).

Supplementary References

1. Kamichi S*, et al.* (2005) Immunohistochemical localization of gastrin-releasing peptide receptor in the mouse brain. *Brain Res* 1032(1-2):162-170.
2. Wada E, Wray S, Key S, & Battey J (1992) Comparison of gene expression for two distinct bombesin receptor subtypes in postnatal rat central nervous system. *Mol Cell Neurosci* 3(5):446-460.
3. Mannino CA, South SM, Inturrisi CE, & Quinones-Jenab V (2005) Pharmacokinetics and effects of 17beta-estradiol and progesterone implants in ovariectomized rats. *J Pain* 6(12):809-816.
4. Gogos A & Van den Buuse M (2004) Estrogen and progesterone prevent disruption of prepulse inhibition by the serotonin-1A receptor agonist 8-hydroxy-2-dipropylaminotetralin. *J Pharmacol Exp Ther* 309(1):267-274.
5. Inami Y, Sasaki A, Andoh T, & Kuraishi Y (2014) Surfactant-induced chronic pruritus: Role of L-histidine decarboxylase expression and histamine production in epidermis. *Acta Derm Venereol* 94(6):645-650.
6. Amaya F*, et al.* (2006) The voltage-gated sodium channel Na(v)1.9 is an effector of peripheral inflammatory pain hypersensitivity. *The Journal of neuroscience : the official journal of the Society for Neuroscience* 26(50):12852-12860.
7. Sakamoto H*, et al.* (2009) Androgen regulates the sexually dimorphic gastrin-releasing peptide system in the lumbar spinal cord that mediates male sexual function. *Endocrinology* 150(8):3672-3679.
8. Satoh K*, et al.* (2015) In vivo processing and release into the circulation of GFP fusion protein in arginine vasopressin enhanced GFP transgenic rats: response to osmotic stimulation. *FEBS J* 282(13):2488-2499.
9. Takanami K*, et al.* (2014) Distribution of gastrin-releasing peptide in the rat trigeminal and spinal somatosensory systems. *The Journal of comparative neurology* 522(8):1858-1873.
10. Andoh T*, et al.* (2017) Prophylactic Administration of Aucubin Inhibits Paclitaxel-Induced Mechanical Allodynia <i>via</i> the Inhibition of Endoplasmic Reticulum Stress in Peripheral Schwann Cells. *Biological and Pharmaceutical Bulletin* 40(4):473-478.
11. Akiyama T, Merrill AW, Carstens MI, & Carstens E (2009) Activation of superficial dorsal horn neurons in the mouse by a PAR-2 agonist and 5-HT: potential role in itch. *The Journal of neuroscience : the official journal of the Society for Neuroscience* 29(20):6691-6699.
12. Funai Y*, et al.* (2014) Systemic dexmedetomidine augments inhibitory synaptic transmission in the superficial dorsal horn through activation of descending noradrenergic control: an in vivo patch-clamp analysis of analgesic mechanisms.

Pain 155(3):617-628.

13. Ohashi N*, et al.* (2017) Acetaminophen Metabolite N-Acylphenolamine Induces Analgesia via Transient Receptor Potential Vanilloid 1 Receptors Expressed on the Primary Afferent Terminals of C-fibers in the Spinal Dorsal Horn. *Anesthesiology* 127(2):355-371.
14. Matsuda KI*, et al.* (2011) Histone deacetylation during brain development is essential for permanent masculinization of sexual behavior. *Endocrinology* 152(7):2760-2767.

Supplementary Table 1

The percentages of GRP (superfusion)- and histamine (i.d.)-responsive neurons

OVX + blank						
Histamine	GRP negative		GRP positive		total	
	n	%	n	%	n	%
negative	7	70.00	9	69.23	16	69.57
positive	3	30.00	4	30.77	7	30.43
total	10	100.00	13	100.00	23	100.00

OVX + estrogen						
Histamine	GRP negative		GRP positive		total	
	n	%	n	%	n	%
negative	11	84.62	4	25.00	15	51.72
positive	2	15.38	12	* 75.00	14	48.28
total	13	100.00	16	100.00	29	100.00

i.d., intradermal injection; OVX, ovariectomy; b, blank; E, estradiol. Chi-square analysis of GRP positive + Histamine positive in OVX + b and OVX + E group; $^*P < 0.01$.

Supplementary Table 2

List of primary antibodies used

Antigen	Immunogen	Species, types	Dilution	Catalog information	RRID
GRP	Rat neuromedin C (or GRP-10; GSHWAVGHLM)	Rabbit polyclonal IgG	1:1,000 (IF)	AssayPro, 11081-05015	AB_2571636
GFAP	Pig GFAP	Mouse monoclonal IgG	1:1,000 (IF)	Sigma-Aldrich, G3893	AB_477010
Iba1	Human Iba1/AIF1	Mouse monoclonal IgG	1:1,000 (IF)	Millipore, MABN92	AB_10917271
NK1R	Rat NK1R (KTMTESSSFYSNMLA)	Rabbit polyclonal IgG	1:5,000 (IF)	Sigma-Aldrich, S8305	AB_261562
c-Fos	Human c-Fos (SGFNADYEASSSRC)	Rabbit polyclonal IgG	1:10,000 (IHC)	Millipore, PC38	AB_2106755
RFP	RFP (clone 8D6)	Mouse monoclonal IgG	1:10,000 (IHC)	MBL, M155-3	AB_1278880
ERα	C-terminus of rat ERα	Rabbit polyclonal IgG	1:2,000 (IF)	Millipore, C1355	AB_310305

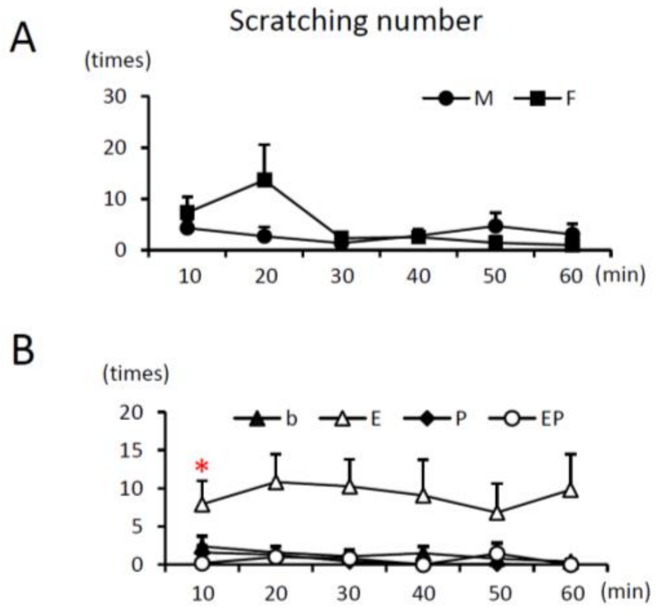

Fig. S1. Sustained histamine-evoked scratching behavior in female OVX rats receiving estradiol replacement.

Graph plots number of scratch bouts elicited by intradermal histamine injection. (**A**) The number of scratching bouts every 10 min of adult male and female rats. (**B**) The number of bouts occurred over a longer duration in female OVX receiving estradiol (OVX + E) groups. In the first 10 minutes, estradiol treatment female showed a significantly higher scratching number than EP treatment (n = 10–11/group, Kruskal Wallis test: H = 10.085, df = 3, P = 0.018; Bonferroni-corrected Mann-Whitney U post-hoc test: U (20) = 20.000, Z = 2.988, P = 0.007, E *vs* EP). Data are mean ± SEM. *P < 0.0083. m, male; f, female; OVX, ovariectomy; b, blank; E, estradiol; P, progesterone; EP, estradiol and progesterone both treatment group.

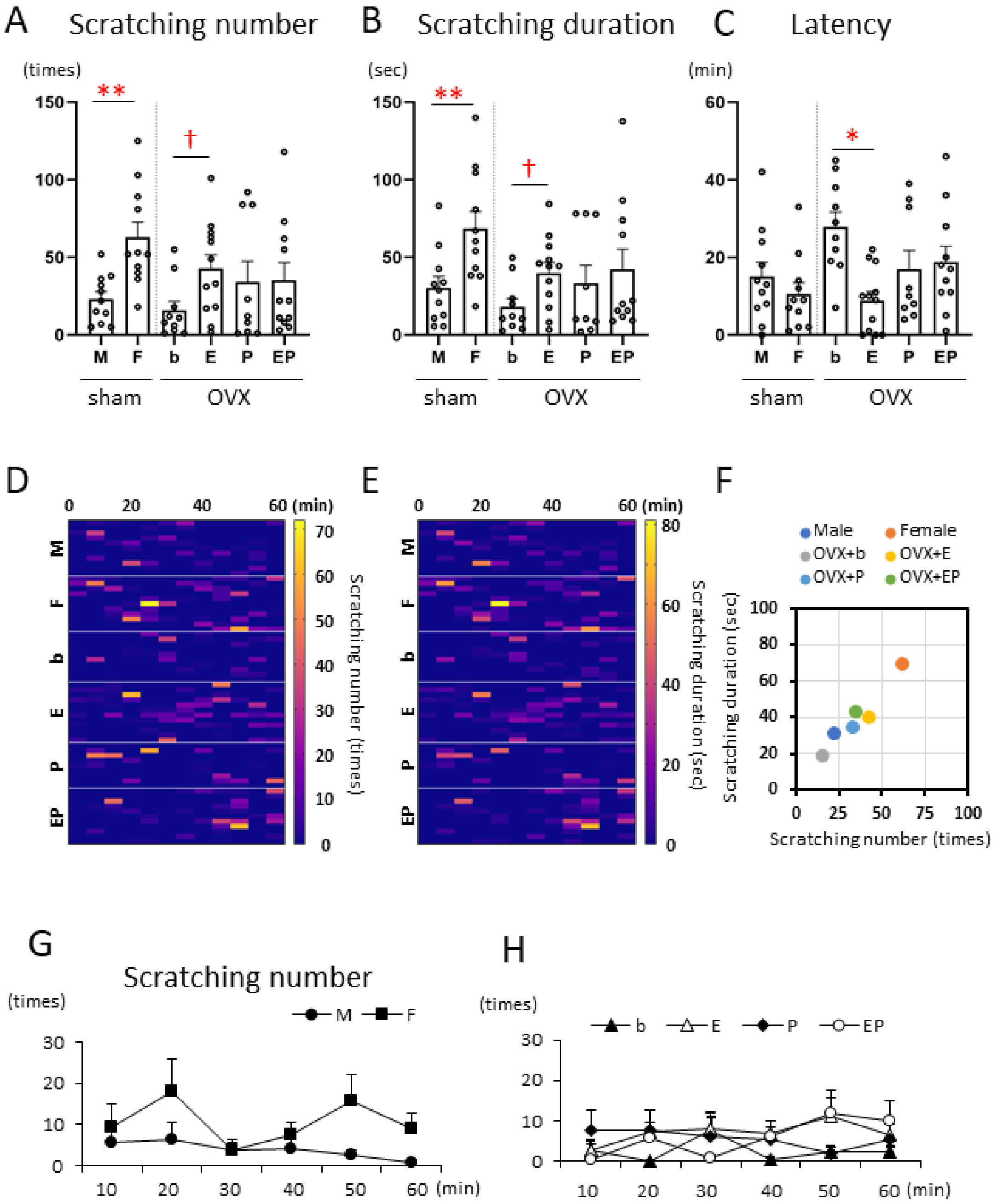
A
Scratching number
(times)
B
Scratching duration
(sec)
C
Latency
(min)
M F b E P EP
sham
OVX
D
E
F
0 20 40 60 (min)
Scratching number (times)
Scratching duration (sec)
Male
Female
OVX+b
OVX+E
OVX+P
OVX+EP
G
Scratching number
M
F
H
b E P EP
10 20 30 40 50 60 (min)

Fig. S2. Chloroquine elicited greater scratching behavior in females than males. Scratching behavior was monitored for 60 min after intradermal injection of chloroquine (10 mg/100 μl). (**A**) The number of chloroquine-evoked scratch bouts was significantly higher in female compared with male rats (*n* = 11/group, Welch's t test: T (14.356) = 3.697, P = 0.002). Among the OVX treatment group, no significant difference was observed (*n* = 9–12/group, Kruskal Wallis test: *H* = 4.829, df = 3, *P* = 0.185). The estradiol-treatment group showed significantly more scratch bouts compared with the OVX + b group (n = 10, 12, respectively, Mann-Whitney *U* test, U (20) = 25.500, Z = 2.276, *P* = 0.021, b *vs* E). (**B**) The duration of chloroquine-evoked scratching was significantly greater in females compared with males (*n* = 11/group, Student's t test: T (20) = 2.884, *P*= 0.009). Among the OVX treatment group, no significant difference was observed (*n* = 9–12/group, Kruskal Wallis test: *H* = 4.237, df = 3, *P* = 0.237). The estradiol treatment group showed a greater duration of scratching compared to the OVX + b group (n = 10, 12, respectively, Mann-Whitney *U* test, U (20) = 28.000, Z = 2.110, *P* = 0.036, b *vs* E). (**C**) There was no sex difference in the latency of chloroquine-evoked scratching (*n* = 11/group, Student's t test: T (20) = 0.974, *P*= 0.341). Among the OVX treatment groups, the estradiol treatment group showed a significantly shorter latency compared with the OVX + b group (*n* = 9–12/group, Kruskal Wallis test: *H* = 11.128, df = 3, *P* = 0.011; Bonferroni-corrected Mann-Whitney U post-hoc test: U (20) = 13.000, Z = 3.105, *P* = 0.001, b *vs* E). (**D**)(**E**) Heat maps plotting the number (D) and duration (E) of scratch bouts/ 5 min elicited by intradermal chloroquine injection for each treatment group. (**F**) A significant correlation was observed between the number of scratch bouts and the total scratching duration/ 60 min in all groups. The value is the average of each group (Pearson's correlation coefficient test: Male, *r* = 0.959, *P* = 0.000; Female, *r* = 0.937, *P* = 0.000; OVX + b, *r* = 0.979, *P* = 0.000: OVX + E, *r* = 0.933, *P* = 0.000; OVX + P, *r* = 0.993, *P* = 0.000; OVX + EP, *r* = 0.993, *P* = 0.000). (**G**)(**H**) Graphs plot number of scratch bouts/ 10 min elicited by intradermal chloroquine injection, for adult male and female rats (G) and OVX treatment groups (H). Data are mean ± SEM. *P < 0.05, **P < 0.01, †P < 0.05. *P < 0.0083 for Bonferroni-corrected Mann-Whitney U post-hoc test following Kruskal-Wallis test among 4 group. M, male; F, female; OVX, ovariectomy; b, blank; E, estradiol; P, progesterone; EP, estradiol and progesterone both treatment group.

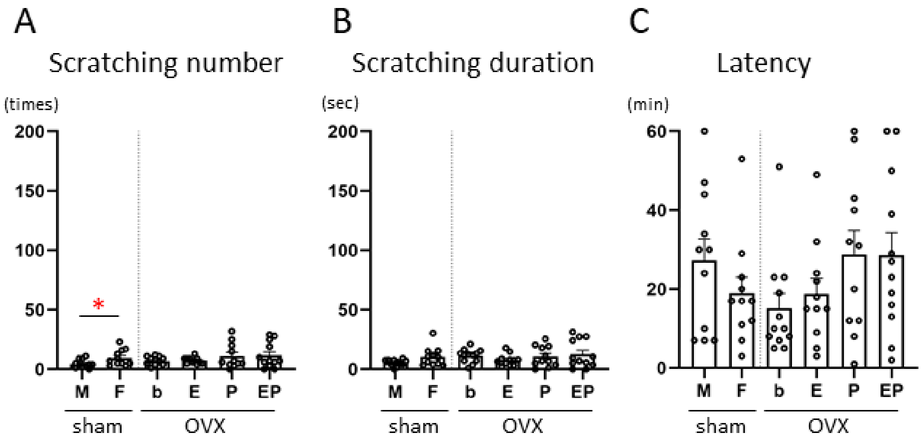

Fig. S3. The estradiol treatment group is not hypersensitive to the skin in the absence of itch stimuli.

Number of scratch bouts (**A**), duration (**B**) or latency of scratching (**C**) following control id injections of saline. (A) Female rats showed the higher scratching number compared with male (n = 11/group, Welch's t test: T (15.266) = 2.194, P = 0.044). Among the OVX treatment group, no significant difference was observed (n = 11–12/group, Kruskal Wallis test: H = 0.508, df = 3, P = 0.917). (B) There was no difference in the scratching duration induced by saline between the groups (Sex difference analysis: Mann-Whitney U test: U (20) = 37.000, Z = 1.543, P = 0.133, OVX group comparative analysis: Kruskal Wallis test: H = 1.740, df = 3, P = 0.628). (C) There was no difference in the latency to scratch between the group (Sex difference analysis: Mann-Whitney U test: U (20) = 44.500, Z = 1.055, P = 0.300, OVX group comparative analysis: Kruskal Wallis test: H = 4.979, df = 3, P = 0.173). Data are mean ± SEM. M, male; F, female; OVX, ovariectomy; b, blank; E, estradiol; P, progesterone; EP, estradiol and progesterone both treatment group.

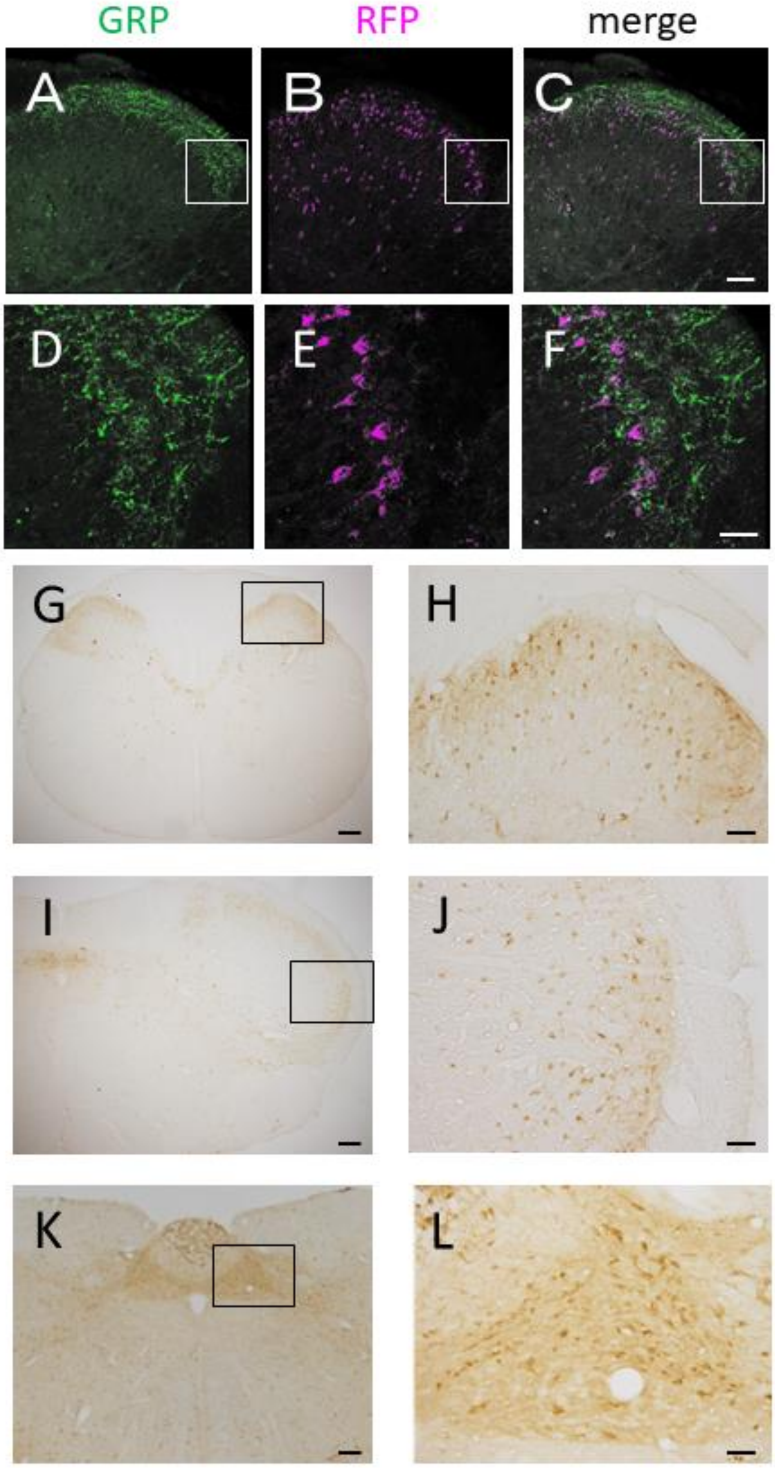
GRP
RFP
merge
A
B
C
D
E
F
G
H
I
J
K
L

Fig. S4. Localization of GRPR-expressing cells labeled with RFP in GRPR-mRFP1 transgenic rats.

(**A–F**) Double-immunofluorescence of GRPR with GRP. GRP immunoreactive terminals (green) (A and D) and GRPR-expressing cells labeled with RFP (magenta) (B and E) are merged in (C and F) to show the localization of GRP/GRPR in the superficial layers of the spinal dorsal horn. Boxed areas in A, B, and C are enlarged in D, E, and F respectively. (**G–L**) GRPR-expressing cells labeled with RFP were localized in the dorsal horn of the spinal cord (G and H), caudal part of the spinal trigeminal nucleus (I and J), and nucleus of the solitary tract (K and L) as previously reported (2). Boxed areas in G, I, and K are enlarged in H, G, and L respectively. Scale bars, 100 μm (A–C); 50 μm (D–F); 200 μm (G, I, K); 50 μm (H, J, L).

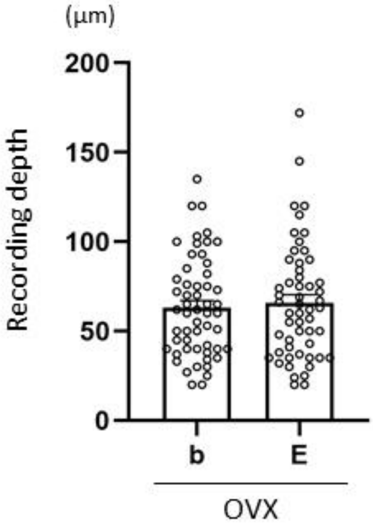

Fig. S5. The depth of the *in vivo* extracellular recordings.

Recording sites were located 20–150 μm below the surface of the spinal cord in OVX + b and OVX + E rats. There was no difference in mean recording depths between the 2 groups (n = 55 and 54 neurons/group, respectively, Mann-Whitney *U* test: U (107) = 1448.500, Z = 0.221, *P* = 0.825). Data are mean ± SEM. OVX, ovariectomy; b, blank; E, estradiol treatment group.

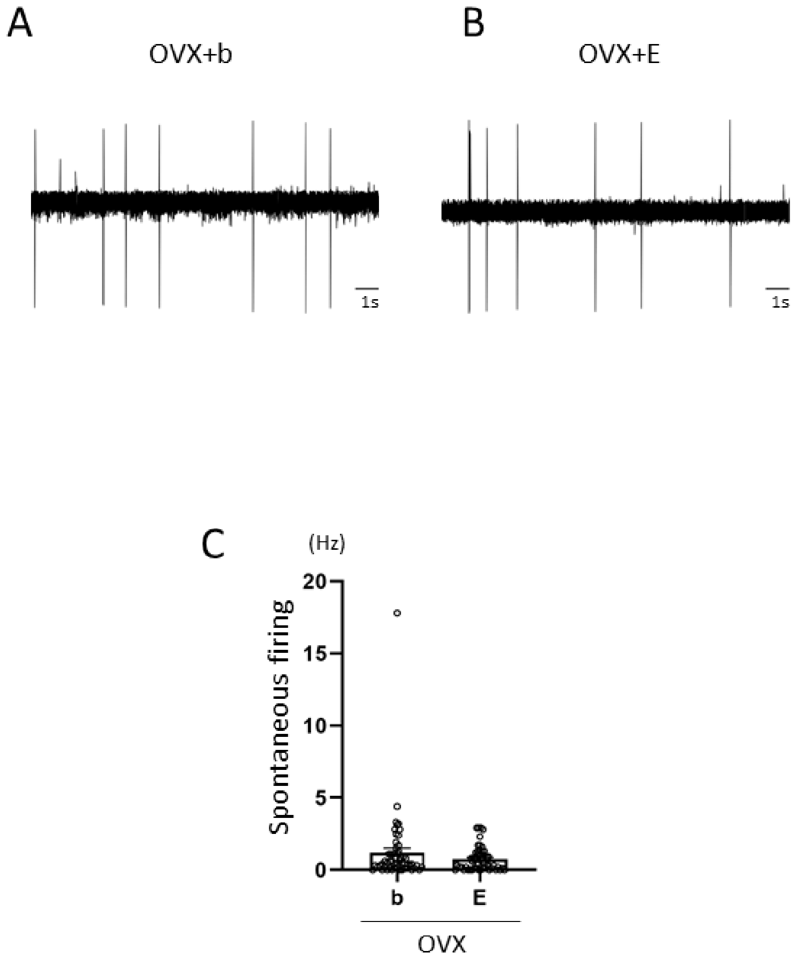

Fig. S6. Spontaneous firing of spinal dorsal horn neurons.
(**A and B**) Representative traces of spontaneous firing of spinal dorsal horn neurons in the OVX + b (A) and OVX + E (B) group. (**C**) There was a low level of spontaneous firing in OVX + b and OVX + E groups (n = 55 and 54 neurons/group, respectively, Mann-Whitney *U* test: U (107) = 1381.000, Z = 0.633, *P* = 0.527). Data are mean ± SEM. OVX, ovariectomy; b, blank; E, estradiol treatment group. Scale bars, 1 sec (A and B).

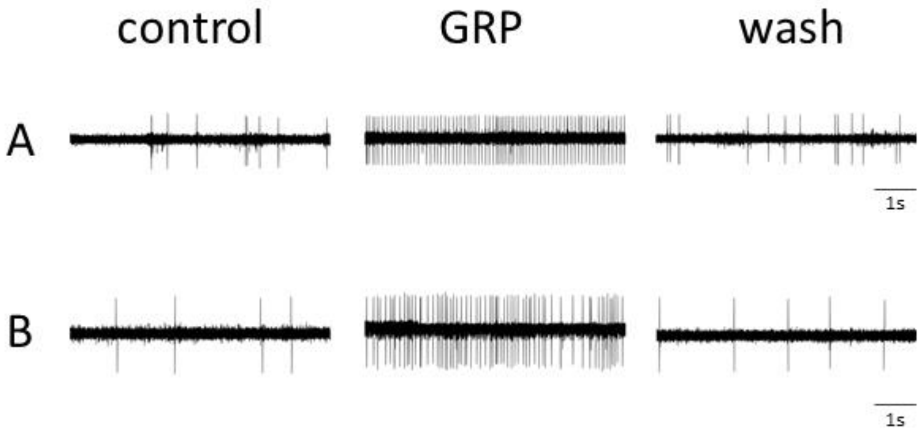

Fig. S7. GRP-evoked firing in the spinal dorsal horn neurons.
(**A and B**) Representative traces of firing induced by superfusion of GRP to the spinal dorsal horn neurons in the OVX + b (A) and OVX + E (B) groups. From the left to right: control (before administration of GRP), GRP superfusion into the spinal dorsal horn, and after wash out. Scale bars, 1 sec (A and B).
